# Dietary fiber induces a fat preference associated with the gut microbiota

**DOI:** 10.1371/journal.pone.0305849

**Published:** 2024-07-10

**Authors:** Yi Jia Liow, Itsuka Kamimura, Masahiro Umezaki, Wataru Suda, Lena Takayasu

**Affiliations:** 1 Department of Human Ecology, School of International Health, Graduate School of Medicine, The University of Tokyo, Bunkyo City, Tokyo, Japan; 2 RIKEN Center for Integrative Medical Sciences, Yokohama, Kanagawa, Japan; 3 Meinig School of Biomedical Engineering, Cornell University, Ithaca, NY, United States of America; Sun Yat-Sen University, CHINA

## Abstract

Eating behavior is essential to human health. However, whether future eating behavior is subjected to the conditioning of preceding dietary composition is unknown. This study aimed to investigate the effect of dietary fiber consumption on subsequent nutrient-specific food preferences between palatable high-fat and high-sugar diets and explore its correlation with the gut microbiota. C57BL/6NJcl male mice were subjected to a 2-week dietary intervention and fed either a control (n = 6) or inulin (n = 6) diet. Afterward, all mice were subjected to a 3-day eating behavioral test to self-select from the simultaneously presented high-fat and high-sugar diets. The test diet feed intakes were recorded, and the mice’s fecal samples were analyzed to evaluate the gut microbiota composition. The inulin-conditioned mice exhibited a preference for the high-fat diet over the high-sugar diet, associated with distinct gut microbiota composition profiles between the inulin-conditioned and control mice. The gut microbiota *Oscillospiraceae sp*., *Bacteroides acidifaciens*, and *Clostridiales sp*. positively correlated with a preference for fat. Further studies with fecal microbiota transplantation and eating behavior-related neurotransmitter analyses are warranted to establish the causal role of gut microbiota on host food preferences. Food preferences induced by dietary intervention are a novel observation, and the gut microbiome may be associated with this preference.

## Introduction

Eating is essential to quality health. The study of eating behavior is an interdisciplinary field that has garnered the attention of nutritionists, anthropologists, psychologists, neuroscientists, sociologists, and even economists, to tackle the central question: *“Why do we eat what we eat?”* Our food choices may seem mundane and arbitrary, yet what we choose to eat has a tremendous effect on our health. Nutritional scientists have illuminated the physiological and metabolic responses elicited by ingested foods’ quantity and composition [1, 2]. Neuroscientists have targeted brain chemistry and neural pathways that control eating behaviors at the core of human behavior [3–5]. Sociologists and psychologists have developed several models to break down and construct a holistic human food selection framework. Anthropologists have shed light on culture’s imprinted effect on food acquisition, preparation, attitudes, and rituals. Each discipline dives into the key question around eating formulated in their own right; however, dysfunctional eating behaviors are increasing drastically in contemporary post-industrial societies [6, 7], unprecedentedly threatening human health.

In nutritional science, two facets of eating behavior, food intake [8] and preference [9–12], have been extensively studied. Food intake is the quantitative measure of ingested food; common parameters include meal size, frequency, and appetite. Food intake regulation has been in the spotlight because obesity, one of the leading causes of deteriorating quality of life, is induced by a chronic positive energy balance. Dietary fiber is one of the most well-studied models for improving food intake to mitigate obesity [13]. Dietary fiber consumption regulates food intake through two mechanisms: (1) it increases satiation and satiety in the host through its physical characteristics [14], and (2) it stimulates the actions of gut-derived hormones on the appetite center in the brain to control food and energy intake [15, 16]. A robust association between dietary fiber intake and eating behavior via the gut-brain axis has been discovered with the recent bloom of gut microbiome studies. Dietary fiber ingestion stimulates the growth of a specific subset of gut microbiota and increases short-chain fatty acid and appetite-related hormone levels [14, 17]. These hormones travel from the gut to the brain and act on the hypothalamus to signal appropriate homeostatic ingestive behavior in the host [16, 18].

Food preference, like food intake, is another key factor in constructing a framework to understand the complexity of human eating behavior. Nutritionists and psychologists have developed instruments to assess food preferences and underlying food motives in human subjects, including the Leeds Food Preference Questionnaire [19] and the Food Choice Questionnaire [20], used across different cultural contexts. Neuroscientists have also conducted extensive interventional studies in animals to examine the neural patterns that serve as the basis of decision-making regarding food, specifically food preferences. The central peptide administration, including neuropeptide Y and opiate in the hypothalamus, elicited a preference for carbohydrate and palatable food consumption [21]. Hormones, including glucagon and growth hormone-releasing hormones, increased protein intake when the mice were offered different macronutrient sources [22–25]. The neurotransmitter serotonin (5-HT) suppresses appetite in high-carbohydrate foods, whereas galanin consistently elicits a preference for high-fat foods [26, 27]. The gut microbiota is responsible for more than 90% of serotonin biosynthesis, a molecule associated with nutrient-specific food preference [28].

Studies targeting direct and indirect food intake control through dietary fiber and gut microbiota have greatly expanded our understanding of eating behavior. However, to the best of our knowledge, no studies have explicitly addressed dietary fiber intake’s impact on food preferences associated with the gut microbiota. Anatomical and physiological similarities between humans and mice make them suitable models for dietary intervention studies. Our study aimed to use a mouse model to clarify dietary fiber consumption’s physiological effects on food preference between palatable high-fat and high-sugar diets in mice associated with the gut microbiota.

## Materials and methods

### Animals and housing

Twelve 8-week-old specific pathogen-free (SPF) inbred C57BL/6NJcl male mice were obtained from CLEA Japan, Inc., Tokyo, Japan. The mice were housed in groups of three, except during the eating behavioral test at the RIKEN Yokohama Campus Animal Facility. They were subjected to a 1-week habituation period and fed a control diet, ad libitum (D21052808, Research Diets, Inc., New Brunswick, NJ, USA). All mice were randomly divided into two groups (inulin-conditioned or control); baseline body weights were measured to ensure the absence of outliers in each experimental group. Ear punching was performed for mouse identification at the beginning of the habituation period. The lights were set to a 12-h light-dark schedule with lights on at 7 a.m. [Zeitgeber (ZT) 0] and off at 7 p.m. (ZT12). The temperature was maintained at 24±1°C and humidity at 50±5% in the SPF animal facility. Animal care and treatment were conducted according to the institutional guidelines of RIKEN Yokohama Campus. The Ethics Committee of the RIKEN Yokohama campus approved all experimental procedures [Y-H29–170187] and complied with the ARRIVE guidelines.

### Dietary intervention

**Fig 1:**
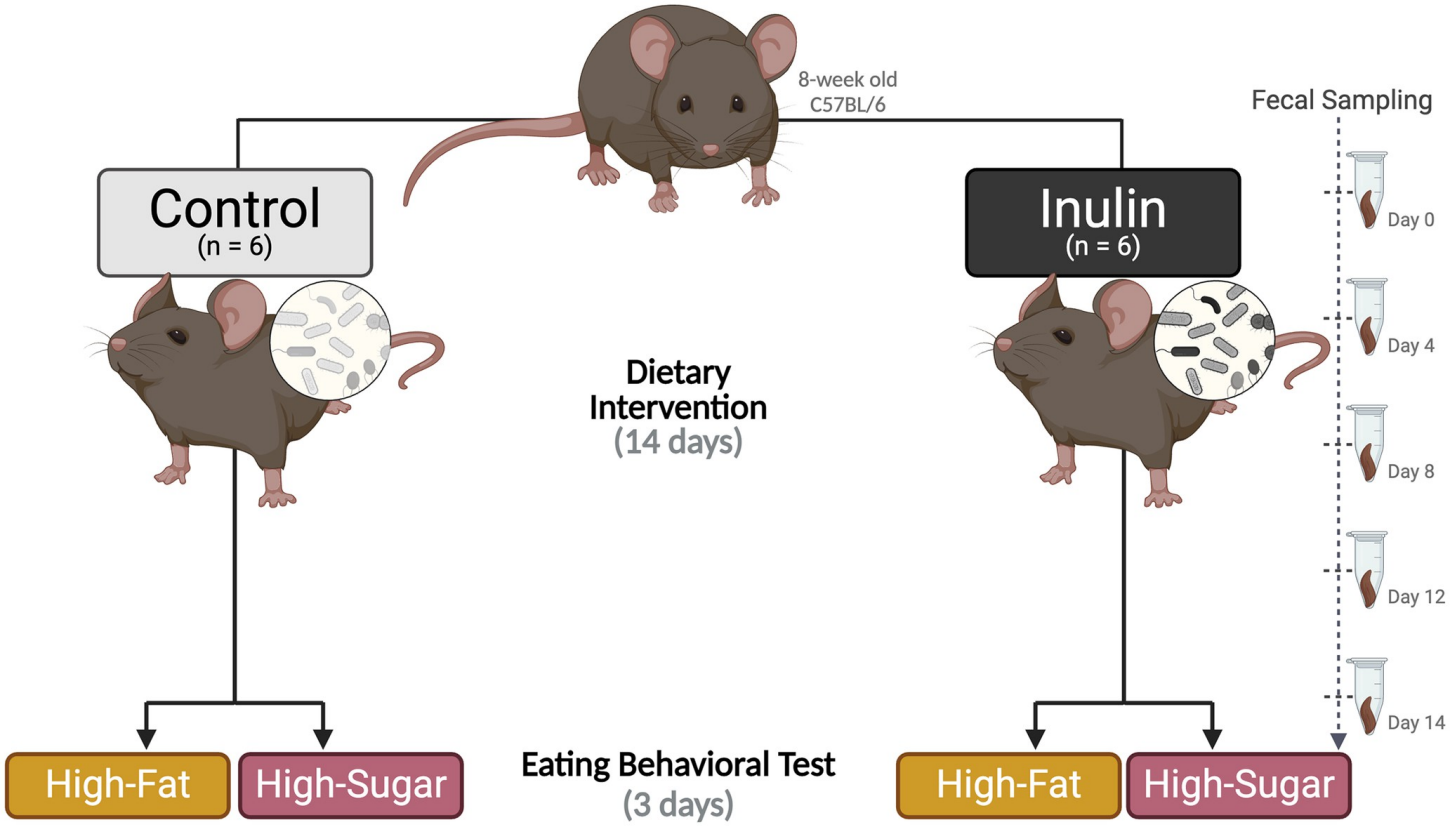
Experimental design overview. Twelve mice underwent a 1-week habituation period followed by a 2-week dietary intervention and were fed one of the following diets: (1) control; (2) high-fiber inulin. After the dietary intervention, the mice were subjected to a 3-day eating behavioral test to choose between palatable high-fat and high-sugar diets. Fecal samples were collected at Day 0 (pre-intervention), 4, 8, 12, 14 (post-intervention), and 17 (post-eating behavioral test). Created with BioRender.com.

At 9 weeks old, all experimental mice were divided into two groups (n = 6/group) and assigned to one of the following feeding regimes for 2 weeks: (a) high-fiber inulin diet (D21052809, Research Diets, Inc., New Brunswick, NJ, USA) or (b) control diet (D21052808, Research Diets, Inc., New Brunswick, NJ, USA), as shown in Fig 1. The control diet, based on AIN-93M, was modified by reducing cellulose content from 5% to 1% to alter gut microbiome composition and short-chain fatty acid production, an alteration associated with shifts in eating behavior [29]. The treatment diet contained approximately the same composition as the control, with 10% inulin added. The control diet comprised 15% protein, 76% carbohydrate, and 4% fat, with an energy density of 4 kcal/g. The high-fiber inulin diet comprised 14% protein, 72% carbohydrate, and 4% fat, with an energy density of 3.88 kcal/g. Both diets’ protein ratio comprised casein (mineral acid 30 mesh) and L-cysteine; the carbohydrate comprised corn starch, maltodextrin 10, sucrose, cellulose, and inulin; the fat comprised soybean oil and t-butylhydroquinone. Table 1 shows a description of the nutritional compositions of the control and treatment diets. The mice’s body weights were measured at the start and end of the dietary intervention.

**Table 1:** Intervention diets nutritional composition.

	Control Diet	Inulin diet
Macronutrient (%)		
Protein	15.00	14.00
Carbohydrate	76.00	72.00
Fat	4.00	4.00
Energy (kcal)	4.00	3.88
Ingredients (gm/3850 kcal)		
Casein	0.00	0.00
Casein, Mineral Acid 30 Mesh	140.00	140.00
L-Cystein	1.80	1.80
Corn Starch	496.00	476.94
Maltodextrin 10	125.00	125.00
Sucrose	100.00	100.00
Cellulose	10.00	10.00
Inulin	0.00	50.00
Soybean Oil	40.00	40.00
t-Butylhydroquinone	0.008	0.008
Mineral Mix S1022M	35.00	35.00
Vitamin Mix V10037	10.00	10.00
Choline Bitartate	2.50	2.50
Source: Research Diets, INC (New Brunswick, NJ)		

### Eating behavioral test

After the 2-week dietary intervention, all experimental mice were housed individually in a customized testing cage (S1 Fig) for a 3-day eating behavioral test to self-select between a palatable high-fat or a high-sugar diet. The testing cage had a 380 × 120 × 115 mm dimension and was made of acrylic materials with three main compartments: two feeding chambers on the left- and right-hand sides and a resting chamber in the middle connecting the feeding chambers. Stainless steel feeding cages were placed on top of the feeding chambers, where the mice could reach the test diets. A stainless-steel lid was placed above the resting area to prevent the mice from escaping and as support for the water bottle. High-fat and high-sugar diets were developed using the AIN-76 rodent diet formulation as the base in collaboration with CLEA Japan, informed by existing literature [27, 30] that highlighted differential dietary preferences within murine models. The high-fat diet comprised 14.9% protein, 44.7% carbohydrate, and 40.4% fat, with an energy density of 4.59 kcal/g. Cocoa butter (20%) was the main fat source. The high-sugar diet comprised 17.4% protein, 70.8% carbohydrate, and 11.8% fat, with an energy density of 4.03 kcal/g. Sucrose was the main sugar source (70%). The test diets’ complete nutritional compositions are listed in Table 2. Their positions were switched every 12 hours to prevent a location bias. The eating behavioral test was conducted during the dark phase, beginning at 7 p.m. and terminating at 7 a.m. the next day. Mice, exhibiting nocturnal tendencies with peak activity and inherent feeding behaviors during nighttime, were assessed for dietary preferences during the dark phase over three consecutive days to precisely capture their natural eating habits during habitual activity periods. The behavioral experiment was carried out in a soundproof room where the temperature was maintained at 24±1°C and humidity at 50±5%. The high-fat and high-sugar test diet feed intakes were recorded at the beginning and end of each 12-h test session. In addition to the test diet feed intake, the fat preference score was calculated as follows:

$$\begin{array}{c} \frac{\text{high-fat[g]}}{\left(\text{high-fat[g]}+\text{high-sugar[g]}\right)} \end{array}$$ (1)

**Table 2:** Test diets nutritional composition.

	High-sugar diet (HS)	High-fat Diet (HF)
Macronutrient (%)		
Protein	17.39	14.92
Carbohydrate	70.79	44.69
Fat	11.82	40.38
Energy (kcal)	4.03	4.59
Ingredients (g)		
Sucrose	70.00	34.90
Cocoa Butter	0.00	20.00
Milk Casein	20.00	19.50
Corn Oil	0.00	0.00
Corn Starch	0.00	15.00
Crystalline Cellulose	5.00	5.00
Mineral Mix (AIN-76)	3.50	3.50
Vitamin Mix (AIN-76)	1.00	1.00
CaCO3	0.00	0.40
DL-Methionine	0.30	0.30
Choline Deltartate	0.20	0.20
Cholesterol	0.00	0.20
3-Butylhydroquinone	0.00	0.004
Source: CLEA Japan, INC.		

The mice were sacrificed by cervical dislocation at the end of the behavioral test.

### Fecal sample collection and bacterial DNA extraction

Fecal samples were collected before, during, and after the dietary intervention to examine the gut microbiota composition changes induced by dietary fiber inulin. Fresh fecal samples were immediately frozen in liquid nitrogen and stored at -80°C until further processing. Fecal pellets were suspended in TE20 and incubated with 15 mg/mL lysozyme (Sigma-Aldrich Co., LLC., St. Louis, MO, USA) and purified achromopeptidase (Wako Pure Chemical Industries, Osaka, Japan) at a final concentration of 100 units/*μ*L at 37°C for 2 h. Furthermore, 1% (w/v) sodium dodecyl sulfate and 1 mg/mL proteinase K (Merck Millipore, Darmstadt, Germany) were added to the fecal pellets and incubated at 55°C for 1 h. The lysate was treated with phenol/chloroform/isoamyl alcohol (NIPPON GENE, Tokyo, Japan). Bacterial DNA was precipitated using 3 M sodium acetate and isopropanol, centrifuged at 12,000 g at 4°C for 5 min, and rinsed with 75% ethanol. RNase (DNAse-free) solution at a final concentration of 10*μ*g/mL was added to the bacterial DNA and incubated at 37°C for 30 min, followed by 20% PEG6000–2.5 M NaCl to precipitate high-molecular-weight DNA by centrifugation at 12,000 g at 4°C for 5 min. The final fecal pellet was rinsed with 75% ethanol three times to remove residual PEG and NaCl, dried under vacuum, and dissolved in 10 mM Tris-HCl/1 mM EDTA.

### 16S rRNA gene amplicon sequencing

The 16S ribosomal RNA (rRNA) gene V1-V2 region was polymerase chain reaction (PCR)-amplified from the bacterial DNA, using the 16S metagenomic sequencing library protocol (Illumina, Inc., San Diego, CA, USA), and amplified using PCR with universal primers 27F-mod (5’-AGRGTTTGATYMTGGCTCAG-3’) and 338R (5’-TGCTGCCTCCCGTAGGAGT-3’). A solution of 44 *μ*L PCR mixture, 2 *μ*L 16S amplicon PCR forward (1 *μ*M) and reverse primers (1 *μ*M), 4 *μ*L bacterial DNA, and PCR-grade water was prepared to a final volume of 50 *μ*L. PCR amplification was conducted with pre-denaturation at 95°C for 3 min, followed by 20 cycles of 95°C for 30 s, 55°C for 30 s, 72°C for 30 s, and a final extension at 72°C for 3 min. The PCR products were purified using AMPure XP beads. Purified products were sequenced using an Illumina MiSeq System (Illumina Inc., San Diego, CA, USA).

### 16S rRNA gene sequencing analysis pipeline

Analysis of the V1-V2 region of the 16S ribosomal RNA began by merging two paired-end reads using fastq-join, focusing on overlapping sequences. Reads with an average quality value below 25, or those not matching both universal primers, were excluded. After trimming the primer sequences, the remaining reads were checked against a reference genome sequence database from the National Center for Biotechnology Information (NCBI) FTP site (ftp://ftp.ncbi.nih.gov/genbank/, Jan 2020). Subsequently, 3,000 high-quality 16S reads with an average quality value exceeding 25 were randomly selected from the filtered reads per sample and trimmed for primers. Low read abundance taxa of less than 0.01% were removed. *α*-diversity was assessed by clustering these reads at a 97% identity threshold, determining the number of OTUs for each sample. *β*-diversity insights were obtained by implementing UniFrac distance analysis, a methodological approach based on quantitative phylogenetic metrics [31].

### Statistical analyses

All statistical tests and data visualizations were performed with R version 4.0.2 and RStudio version 1.3.1093. The Wilcoxon rank-sum test was used to test for statistical differences in (1) the high-fat and high-sugar test diet feed intake, (2) the high-fat and high-sugar test diet energy intake, and (3) body weight changes among the inulin-conditioned and control mice. The MaAsLin2 package was used to examine the differences in gut microbiota composition between the control and inulin-conditioned groups at three critical time points: before the intervention (Day 0), after the intervention (Day 14), and after the preference test (Day 17). Permutational multivariate analysis of variance (PERMANOVA) was performed using the adonis function of the vegan package to identify community-level differences between groups, with adjustment for multiple testing using the False Discovery Rate (FDR) q-value correction method. Spearman’s rank correlation tests were applied to the gut microbiota data collected on Day 14, in conjunction with the calculated fat preference scores. Subsequently, these correlations were adjusted using the False Discovery Rate (FDR) q-value method to identify microbial taxa that exhibited statistically significant associations with fat preference.

## Results

### Dietary fiber consumption and nutrient-specific food selection

**Fig 2:**
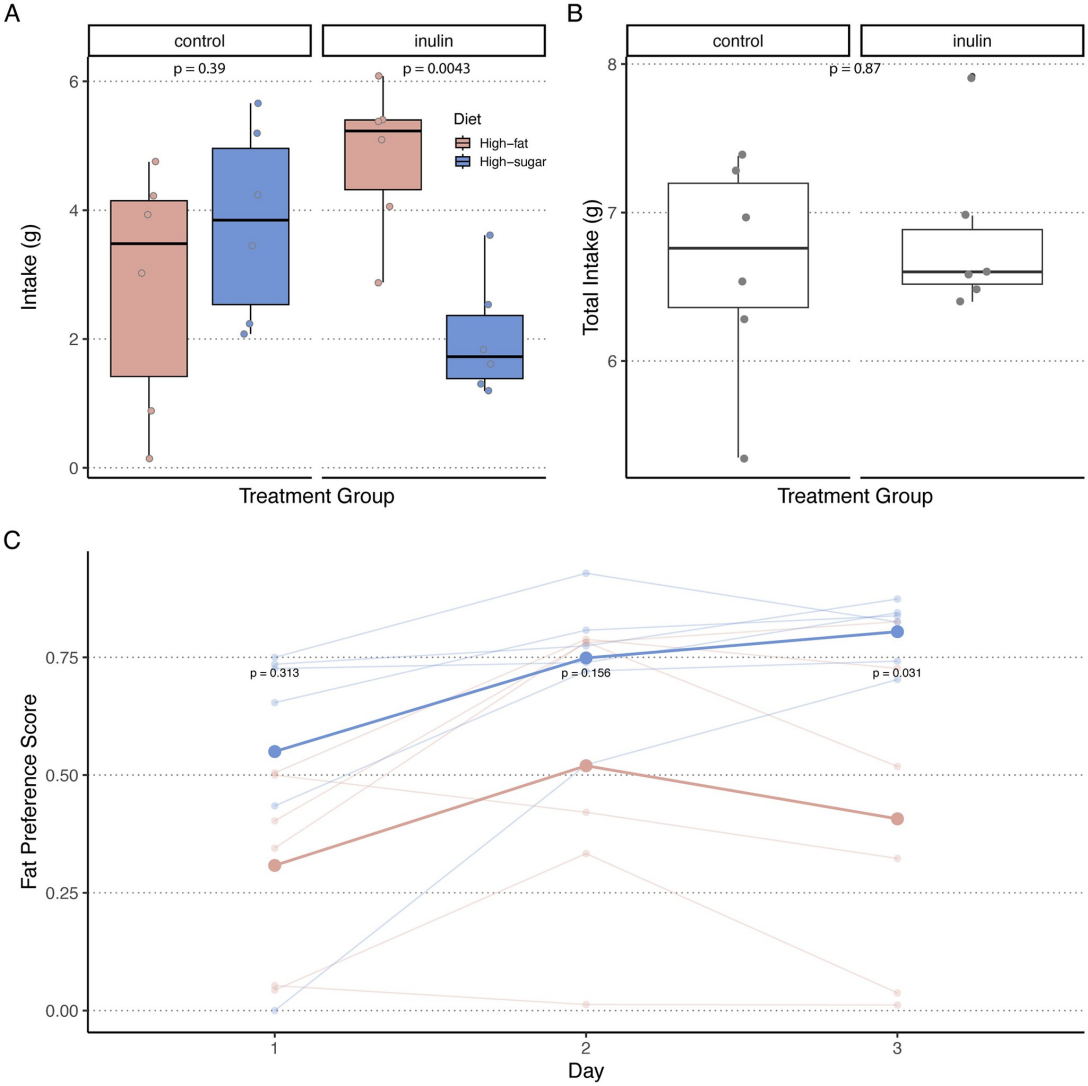
Nutrient-specific food selection results. Fig 2A shows a significant difference in the high-fat and high-sugar feed intakes in inulin-conditioned mice (P = 0.0043); however, no significant difference was detected in the control group (P = 0.39). Fig 2B shows no significant differences in the total test diet intake between the two groups (P = 0.87). Fig 2C shows the fat preference scores of the inulin-conditioned and control mice over the course of the three-day eating behavior test. The inulin-conditioned mice showed a significant increase in fat preference on Day 3 (P = 0.031).

To investigate the influence of dietary fiber inulin on food preferences, our experiment subjected mice to a two-choice food preference test, which revealed a marked preference for high-fat over high-sugar diets after a two-week inulin dietary intervention (Fig 2). Fig 2 compares the high-fat and high-sugar test diet intakes, including the total feed intake between the two mice groups. Fig 2A shows that the inulin-conditioned mice significantly preferred the high-fat diet (P = 0.0043) over the high-sugar diet, whereas no significant difference in preference was detected in the control mice (P = 0.39). Moreover, high-fat and high-sugar diet intake was compared between the two groups. Considering the drastic difference in the test diets’ macronutrient proportions, the inulin-conditioned and control mice’s combined feed intakes (high-fat + high-sugar) were compared. However, no significant differences in total feed intake were detected (P = 0.87), as shown in Fig 2B. We also computed the fat preference scores in the inulin-conditioned and control mice (Fig 2C). An increase in fat preference was observed over the course of the three-day eating behavior test, culminating in a pronounced difference in mean fat preference between the two groups on Day 3 (P = 0.031). These results show that inulin-conditioned mice prefer a high-fat diet with a suppressed appetite for a high-sugar diet. Furthermore, when comparing the caloric intake of the high-fat and high-sugar diets (S2 Fig), a significant difference was observed in the inulin-conditioned mice, aligning with the food preference test results depicted in grams (P = 0.0043). However, no significant differences were detected in the total caloric intake between the two groups (S2 Fig). The inulin-conditioned and control mice’s body weights increased significantly after dietary intervention (P = 0.0026 and 0.0045, respectively, (S3 Fig)). Nonetheless, there was no significant difference in the percent change in body weight between the two groups of mice before and after the dietary intervention (P = 0.13).

### Taxonomical features of gut microbiota post-intervention and preference test

**Fig 3:**
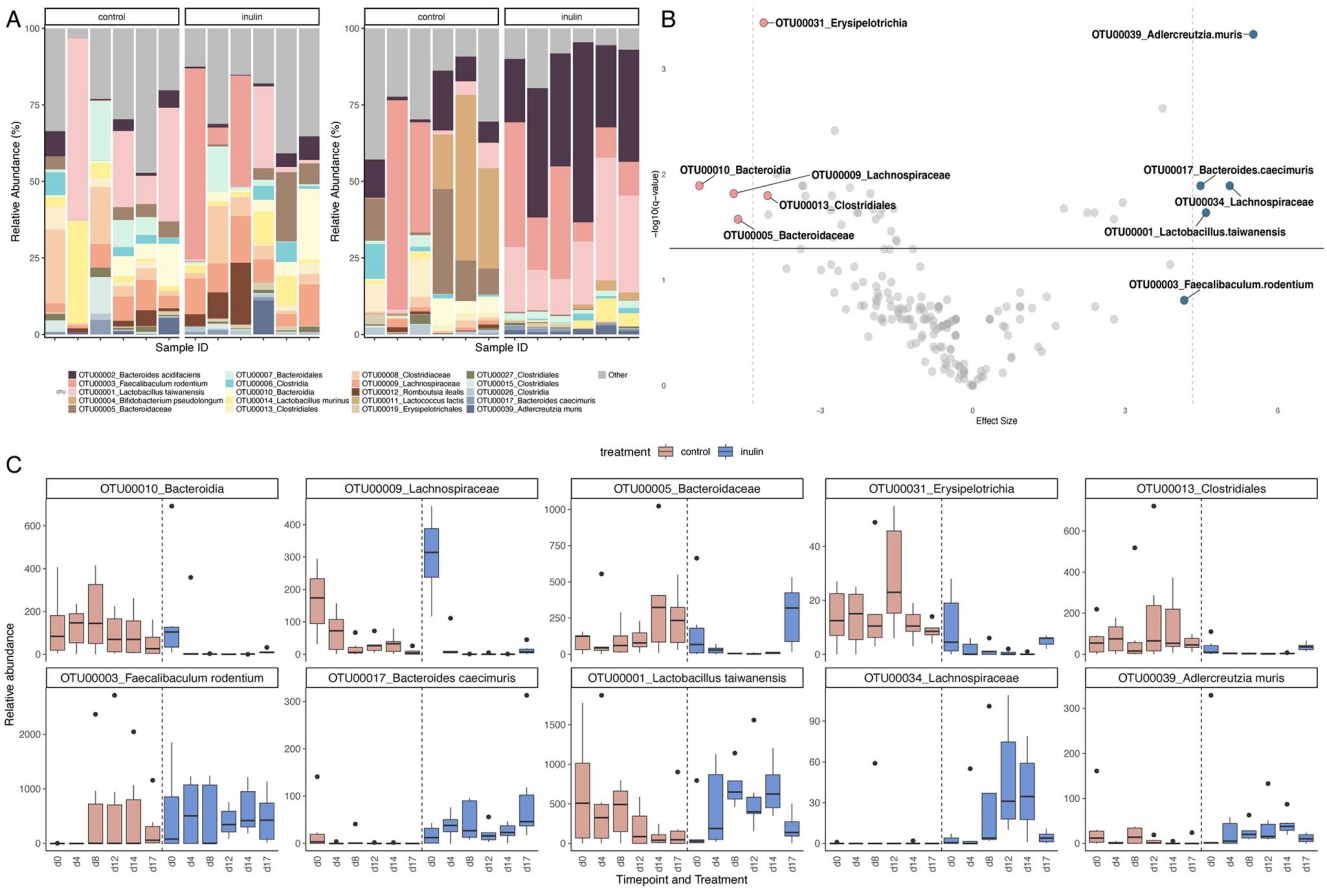
Taxonomical features of gut microbiota post-dietary intervention. Fig 3A: OTU-level taxonomic composition of the control and inulin-conditioned mice pre-intervention and post-intervention; Fig 3B: Volcano plot of the MaAsLin2 model output, showing the top 10 taxa that were significantly enriched or depleted in the inulin-conditioned mice compared to the control group; Fig 3C: Longitudinal dynamics of the top 10 taxa showing significant differences in abundance between the two groups.

To investigate the impact of a two-week dietary intervention with standard control and high-fiber inulin diets on the gut microbiota, we analyzed fecal pellets from mice using the 16S rRNA gene amplicon sequencing and uncovered significant alterations in the gut microbiota profile after the intervention and following the food preference test (Fig 3). MaAsLin2 analysis revealed a total of 123 OTUs that showed significant differential abundance when comparing the control group to the inulin-conditioned mice. Fig 3A illustrates the OTU-level taxonomic composition. In addition, phylum- and family-level analyses complemented these results, revealing notable shifts in key bacterial phyla such as Firmicutes and Bacteroidetes, as well as in specific families (S4 and S5 Figs). Fig 3B features a volcano plot which delineates the top 10 taxa which experienced the most pronounced enrichment or depletion as a result of the inulin treatment throughout the intervention. The taxa that were most significantly enriched include *Adlercreutzia muris* (OTU00039), *Bacteroides caecemuris* (OTU00017), *Lachnospiraceae* (OTU00034), *Lactobacillus taiwanensis* (OTU0001), and *Faecalibaculum rodentium* (OTU0003). Conversely, the taxa that underwent the most substantial depletion were *Erysipelotrichia* (OTU00031), *Bacteroidia* (OTU00010), *Clostridiales* (OTU00013), *Bacteroidaceae* (OTU0005), and *Lachnospiraceae* (OTU0009). To elucidate the temporal dynamics of the top 10 taxa showing significant differences between the two groups, Fig 3C provides a longitudinal depiction of the fluctuations in abundance of each taxon at specific time points (Day 0, 4, 8, 12, 14, 17). The top row of the figure shows taxa that were enriched, while the bottom row highlights those that were depleted. A heatmap based on hierarchical clustering was generated to visualize the bacterial composition dynamics after dietary intervention (S6 Fig).

Significant differences were observed in 13 OTUs as a result of the three-day food preference test, in which mice were free to choose between a high-fat and a high-sugar diet. The analysis revealed significant shifts in the microbial landscape that were annotated in a volcano plot (S7 Fig). The plot shows the top 10 significantly enriched bacterial taxa after the test were *Firmicutes* (OTU00081), *Ruminococcaceae* (OTU00082), *Clostridia* (OTU00026), *Bacteroides caecemuris* (OTU00017), *Clostridiales* (OTU00027 and OTU00221), *Faecalimonas sp*. (OTU00106), *Muribaculum intestinale* (OTU00037), *Lacnospiraceae* (OTU00045), and another distinct lineage of *Ruminococcaceae* (OTU00205). In addition to identifying these enriched taxa, longitudinal observations mapped changes in their relative abundance over time, indicating dynamic changes in the microbiota in response to experimental diets. Shannon diversity index showed no significant change in total microbial diversity after the preference test (S8 Fig). *β*-diversity analysis using weighted UniFrac also showed no significant difference, with an R2 value of 0.08 and a p-value of 0.487 (q = 0.487), indicating that the overall community composition did not differ significantly between groups in response to the high-fat and high-sugar test diets (S9 Fig). These results suggest that while certain taxa showed significant enrichment, the overall microbial community structure remained relatively unaffected over the course of the food preference test.

The Shannon diversity index computation for *α*-diversity revealed no significant differences in microbial community richness and evenness between the control group and inulin-conditioned mice at pre-intervention, post-intervention, and post-preference test phases. In contrast, *β*-diversity assessed using the weighted UniFrac distance demonstrated divergence between the groups at the post-intervention stage. Principal coordinates analysis (PCoA) attributed 28.69% of the total variance to PCoA1 and 27.41% to PCoA2, resulting in an R2 value of 0.27, with a statistically significant p-value of 0.016 and a q-value of 0.048. Conversely, the pre-intervention and post-preference test phases presented no significant differences in microbial community composition between the groups, recording R2 values of 0.107 (P = 0.312, q = 0.468) and 0.08 (P = 0.487, q = 0.487), respectively. These results highlight the capacity of inulin treatment to alter microbial community structure, with the effects predominantly observable post-intervention through the *β*-diversity metrics.

### Correlation between gut microbiota and nutrient-specific food preference

**Fig 4:**
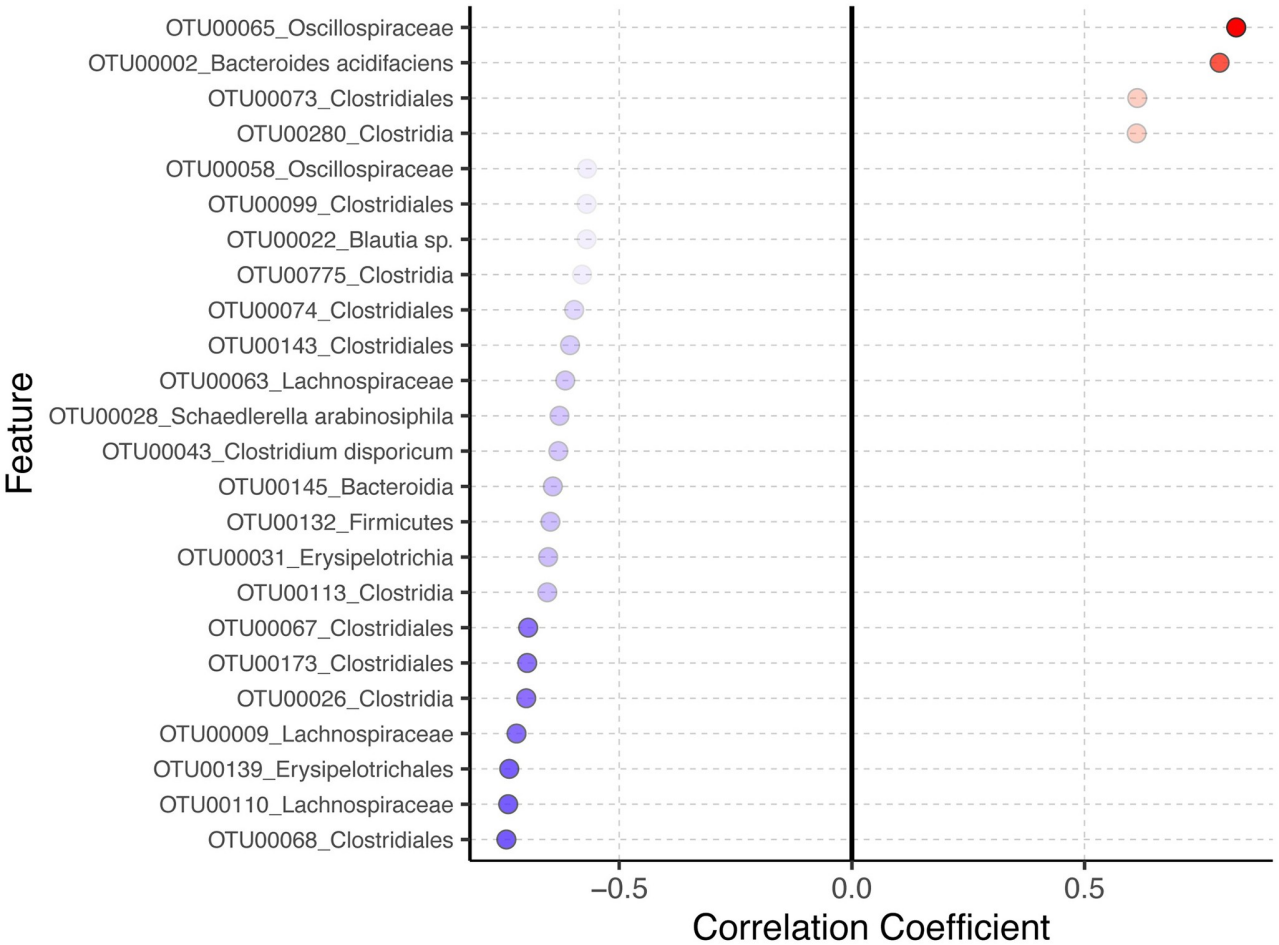
Correlation between fat preference and gut microbiota. 24 OTUs significantly correlated with fat preference. Four out of 24 OTUs positively correlated with fat preference: OTU00065 *Oscillospiraceae*, OTU00002 *Bacteroides acidifaciens*, OTU00073 *Clostridiales*, and OTU00280 Clostridia (q = 0.0413, 0.0562, 0.0849, and 0.0849, respectively.) The size and color intensity of the dots represent the strength of the correlation.

To explore the correlation between gut microbiota composition and dietary preferences, we employed the computed fat preference scores and revealed distinct microbial correlations with preference for fat. In Fig 4, 24 OTUs are correlated with hedonic eating patterns, particularly fat preference. Four OTUs exhibited a positive correlation with fat preference: OTU00065 from the *Oscillospiraceae* family (q = 0.0413), OTU00002 identified as *Bacteroides acidifaciens* (q-value = 0.0562), and OTU00073 and OTU00280, both from the *Clostridiales* order (q = 0.0849). Conversely, 20 OTUs showed a negative correlation with fat preference, suggesting a preference for sugar. Notable mentions include OTUs from the *Lachnospiraceae* family (OTU00110, OTU00009, OTU00063) and a range of OTUs under the *Clostridiales* classification (OTU00068, OTU00026, OTU00173, OTU00067, OTU00113, OTU00143, OTU00074, OTU00775, OTU00099) with q-values between 0.0593 to 0.0991. Additionally, OTUs such as OTU00139 (*Erysipelotrichales*), OTU00031 (*Erysipelotrichia*), OTU00132 (*Firmicutes*), OTU00145 (*Bacteroidia*), OTU00043 (*Clostridium disporicum*), OTU00028 (*Schaedlerella arabinosiphila*), and OTU00022 (*Blautia sp*.) were also observed with similar q-values. This analysis highlights the relationship between microbial composition and dietary preferences.

## Discussion

Food preference is an essential component to maintaining health status in addition to cumulative food intake. The present study demonstrated that consumption of soluble dietary fiber inulin altered gut microbiota composition and induced a preference for a high-fat over a high-sugar diet. Existing literature has extensively discussed the benefits of dietary fiber on controlling food intake.

To date, this is the first study that examines the impact of dietary fiber inulin consumption on food preference between palatable high-fat and high-sugar diets. Dietary fiber inulin is known to control food intake and regulate one’s appetite, as commonly determined by total energy or cumulative food intake [32]. Yet the effect of inulin on food preference has not been studied. Our results show that consuming dietary fiber inulin induces a preference for fat and suppresses the appetite for sugar in a mouse model. This observation suggests that previous dietary consumption substantially affects subsequent food choices. Nutrient-specific food selection operates on a positive feedback mechanism [30] in which pre-exposure to a certain macronutrient induces a preference for that macronutrient in mice. For example, the mice pre-exposed to fat self-selected fat, protein self-selected protein, and carbohydrates self-selected carbohydrates. A similar study demonstrated a contradictory result in which high pre-meal protein composition induced a low protein intake in subsequent meals with a reduction in total food intake [33]. Regardless, evidence demonstrates that previous meals substantially influence subsequent meals’ quantity and composition. Our results support the existing literature that the composition of the previous meal determines subsequent nutrient-specific food selection, even when the test diet compositions are not presented in the preceding meal. Preceding dietary composition’s effect on subsequent food choice was investigated more than three decades ago without consideration of gut microbiota as a component in the framework [33, 34]. Our study is one of the first that reinforces the effect of preceding dietary composition on subsequent food selection, considering the gut microbiota as a factor.

Soluble dietary fiber consumption controls experimental animals’ energy intake and body weight [35, 36], although several studies have reported conflicting results. For example, some studies have reported that short-term inulin supplementation (12 days to 4 weeks) does not control body weight gain in experimental mice compared with controls [37–39]. In the present study, significant weight gain was detected in the inulin-conditioned and control mice after the 2-week inulin intervention; no significant differences were detected in the post-dietary intervention body weights between the two groups. In particular, the mice in the present study were still undergoing their growth phase during the dietary intervention period (PD64–80), explaining the significant weight gain after dietary intervention. Several studies have reported that soluble dietary fiber increases metabolizable energy extraction from feed compared with insoluble dietary fiber, increasing body weight and leading to the development of the obese phenotype in mice [40, 41]. In the present study, we observed a trend toward greater weight gain in inulin-conditioned mice compared to the control mice. However, the lack of bomb calorimetry data limits our ability to directly link changes in gut microbiota and associated energy balance, as dietary differences could inherently affect energy expenditure and consequently body weight. Determination of the energy content of collected fecal pellets may be used to substantiate this observation in the future.

We compared nutrient-specific food selection in mice between two palatable test diets: a high-sugar diet (carbohydrate, 70.79%; fat, 11.82%; protein, 17.39%) and a high-fat diet (carbohydrate, 44.60%; fat, 40.38%; protein, 14.92%). Obesity results from a chronic energy imbalance between energy intake and expenditure, and food addiction exacerbates its development. The food addiction model is explained by neural activation in the reward circuitry in response to food cues and reduced activity in the inhibitory regions following palatable food intake. Several reports have demonstrated that sugar is a food component that causes addictive behavior [42–46]. In contrast, while fat consumption does not elicit addictive behavior, its overconsumption contributes to excessive weight gain from fat mass accumulation [42, 47, 48]. High-fat and high-sugar foods are ubiquitous in the contemporary food environments and have differential downstream physiological effects when ingested [49]. Food addiction is problematic because, unlike drugs and alcohol [4, 50], humans cannot eliminate food as it sustains survival. The present study assessed dietary fiber consumption’s effect on subsequent nutrient-specific food selection between palatable high-fat and high-sugar diets. Dietary fiber consumption regulates food and energy intake quantity and promotes a preference for fat over sugar, curbing food addiction development.

In the present study, three bacterial species positively correlated with fat preference: Oscillospiraceae sp., *Bacteroides acidifaciens*, and *Clostridiales sp*. These bacterial species were enriched in response to dietary fiber inulin consumption, consistent with several reports [51–53]. A recent study reported that the genus *Bacteroides*, particularly *Bacteroides uniformis CECT 7771*, is involved in reducing binge-eating behavior in rats, a process mediated by the serotonergic and dopaminergic pathways in the hypothalamus [54]. Another study revealed an inverse correlation between the relative abundance of *Bacteroides* and addiction-like eating behavior in obese women who underwent laparoscopic sleeve gastrectomy by reducing connectivity in the brain reward regions [55]. Combining the results from the present study illustrating that *Bacteroides* are positively correlated with a preference for a high-fat over a high-sugar diet, our findings imply *Bacteroides*’ protective role against addiction-like eating behavior in human and animal subjects. This study, however, is limited by the absence of fecal transplantation experiments, which are essential to establish a causal relationship between gut microbiota composition and the observed changes in food preferences. Further studies involving fecal microbiota transplantation onto germ-free mice and measurement of candidate neurotransmitters are necessary to draw a causal relationship between gut microbiota composition and food preference.

Existing studies have elucidated how diet influences the gut microbiota. Specifically, the increase in bacterial groups such as *Clostridiales* and *Erysipelotrichales* in our study parallels the findings of Magnusson et al. where these bacteria increased in mice consuming a 62% high sugar diet [56]. However, the increase in *Lachnospiraceae* that we observed contrasts with previous research showing a decrease with consumption of a high-sugar diet [57]. This inconsistency underscores the potential for unique interactions between diet type, microbial composition, and other factors, and highlights the need for more nuanced research. Previous literature has identified a relationship between the gut microbiome and brain connectivity, notably within the reward network involving regions such as the putamen and precuneus [54]. Dong et al. pointed out that a higher abundance of *Lachnospiraceae* was associated with low connectivity between these brain regions. Our current findings further delve into the implications of such microbial patterns on dietary preferences. We found that an increase in *Lachnospiraceae* was significantly correlated with a negative fat preference. These correlations, taken together, suggest a possible link wherein the abundance of specific microbial taxa, such as *Lachnospiraceae*, may not only influence brain connectivity patterns but also modulate dietary preferences. This further emphasizes the intricate intertwining of gut-brain interactions in determining food choices. The genus *Bacteroides* has previously been implicated in feeding behavior. For example, administration of *Bacteroides uniformis* to rats altered their binge eating behavior and affected the brain’s reward response [53]. In parallel with this literature, our study found that *Bacteroides acidifaciens*, another species within the *Bacteroides* genus, showed a positive correlation with fat preference. This consistent association across studies highlights the potential role of *Bacteroides* in the modulation of food preferences.

The current findings, demonstrating significant shifts in 13 OTUs after a three-day food preference test in which mice chose between high-fat and high-sugar diets, dovetail with existing literature on the subject. Suriano et al. underscore that diet-induced obesity is closely tied to deviations in both the composition and functionality of the gut microbiota. It further illustrates that diets high in sugar and fat can differentially impact the microbiota, subsequently affecting obesity and related comorbidities [58]. In line with the literature, our results suggest that the significant enrichment of specific bacterial taxa, such as Firmicutes, Ruminococcaceae, and Bacteroides, may be a reflection of the microbiota’s response to the macronutrient composition of the diet. This study also extends previous research indicating that changes in the gut microbiota can lead to increased intake of palatable foods through the microbiota-gut-brain axis [59]. It examines the influence of inulin, a dietary fiber, on these microbial communities and subsequent food preferences. A notable preference for high-fat diets in mice supplemented with inulin suggests that dietary components can significantly shape gut microbiota, which in turn guides food choices. This finding suggests that dietary interventions could reprogram eating behaviors associated with overeating disorders. The research not only strengthens the literature, but also broadens the understanding of diet-microbiome interactions and presents potential new therapeutic avenues within the microbiota-gut-brain framework.

## Conclusions

In summary, we demonstrated that dietary fiber consumption may induce a preference for a high-fat diet over a high-sugar diet, associated with the enrichment of gut bacteria *Oscillospiraceae*, *Bacteroides acidifaciens*, and *Clostridiales*. Our data on the impact of dietary fiber inulin on food preference may contribute to understanding the behavioral consequences of dietary fiber ingestion on future food preferences through gut microbiota modulation.

## Supporting information

S1 Fig Customized eating behavioral testing cage. The left- and right-chambers are the feeding chambers for mice to self-select between the test diets; the central area is the resting area for when the mice are not interacting with food. Marble balls are modifications made to the cage to prevent the mice from resting in the feeding chambers; stainless steel clips are used to secure the lid to prevent the mice from escaping.
(TIF)

S2 Fig Energy intake comparison between the inulin-conditioned and control mice. Fig 3A: No significant differences were detected in the energy intake of high-fat and high-sugar test diet energy intakes among the inulin-conditioned and control mice (P = 0.7 and 0.13, respectively). Fig 3B: Total energy intake combining high-fat and high-sugar test diets of the inulin-conditioned and control mice; no significant differences were detected in the total energy intake between the two groups (P = 0.66).
(TIF)

S3 Fig Effects of inulin treatment on body weight in mice. The left panel illustrates the body weight of mice from control and inulin-conditioned groups at two distinct time points: pre-intervention (baseline) and post-intervention. Each line represents the weight trajectory of an individual mouse. The right panel visualizes the percentage change in body weight for each group. The difference in weight gain between the two groups was not statistically significant (P = 0.229).
(TIF)

S4 Fig Comparative analysis of phylum-level taxonomic composition in control and inulin-conditioned groups. The figure illustrates the relative abundance of different bacterial phyla within each sample group (control and inulin-conditioned) at both the pre- and post-intervention stages, highlighting shifts in microbial diversity in response to the intervention.
(TIF)

S5 Fig Comparative analysis of family-level taxonomic composition in control and inulin-conditioned groups. The figure illustrates the relative abundance of different bacterial phyla within each sample group (control and inulin-conditioned) at both the pre- and post-intervention stages, highlighting shifts in microbial diversity in response to the intervention.
(TIF)

S6 Fig Heatmap of the significantly different OTUs between the inulin-conditioned and control mice post-dietary intervention (Day 14) based on hierarchical clustering. The colors on the heatmap reflect the log-transformed OTU relative abundance; red indicates OTUs high in relative abundance and blue indicates OTUs low in relative abundance.
(TIF)

S7 Fig Differential relative abundance of bacterial taxa post-preference test. S5A Fig shows a volcano plot showcasing bacterial taxa that exhibited significant differences in relative abundance following a preference test, as analyzed by MaAsLin2. The top 10 taxa with the most pronounced differences are highlighted with labels. Supplementary S5B Fig provides a longitudinal assessment of the relative abundance of these top taxa at distinct time points (Days 0, 4, 8, 12, and 14).
(TIF)

S8 Fig *α*-diversity analysis. S6 Fig illustrates the *α*-diversity (Shannon diversity index) of microbial communities for the control and inulin-conditioned groups at three distinct time points: pre-intervention, post-intervention, and post-preference tests were not significantly different between the control and inulin-conditioned mice (P = 0.94, 0.065, 0.48, respectively).
(TIF)

S9 Fig *β*-diversity calculated with weighted UniFrac distances. S7 Fig showcases microbial community dissimilarity during three stages: pre-intervention, post-intervention, and post-test. Gut microbiota profiles were significantly different between the control and inulin-conditioned mice at the post-intervention time point (R2 = 0.27, q-value = 0.048), but not at the pre-intervention (R2 = 0.107, q-value = 0.468) or post-preference test (R2 = 0.08, q-value = 0.487) time points.
(TIF)

## References

[1] Popkin BM, Adair LS, Ng SW. Global nutrition transition and the pandemic of obesity in developing countries. Nutrition Reviews. 2012;70(1):3–21. doi: 10.1111/j.1753-4887.2011.00456.x 22221213 PMC3257829

[2] Kim H, Hu EA, Rebholz CM. Ultra-processed food intake and mortality in the USA: results from the Third National Health and Nutrition Examination Survey (NHANES III, 1988–1994). Public Health Nutrition. 2019;22(10):1777–1785. doi: 10.1017/S1368980018003890 30789115 PMC6554067

[3] Gearhardt A N, Davis C, Kuschner R, Brownell K D. The Addiction Potential of Hyperpalatable Foods. Current Drug Abuse Reviewse. 2011;4(3):140–145. doi: 10.2174/1874473711104030140 21999688

[4] Blumenthal DM, Gold MS. Neurobiology of food addiction:. Current Opinion in Clinical Nutrition and Metabolic Care. 2010;13(4):359–365. doi: 10.1097/MCO.0b013e32833ad4d4 20495452

[5] Fletcher PC, Kenny PJ. Food addiction: a valid concept? Neuropsychopharmacology. 2018;43(13):2506–2513. doi: 10.1038/s41386-018-0203-9 30188514 PMC6224546

[6] Santana DD, Mitchison D, Griffiths S, Appolinario JC, Veiga GV, Touyz S, et al. Ten‐year time trends in mental and physical health correlates of weight/shape overvaluation. European Eating Disorders Review. 2019; p. erv.2672. doi: 10.1002/erv.2672 30895707

[7] Tsai M, Gan S, Lee C, Liang Y, Lee L, Lin S. National population‐based data on the incidence, prevalence, and psychiatric comorbidity of eating disorders in Taiwanese adolescents and young adults. International Journal of Eating Disorders. 2018;51(11):1277–1284. doi: 10.1002/eat.22970 30488563

[8] Westerterp-Plantenga M. Eating behavior in humans, characterized by cumulative food intake curves—a review. Neuroscience & Biobehavioral Reviews. 2000;24(2):239–248. doi: 10.1016/S0149-7634(99)00077-9 10714387

[9] Bauer F, Elbers CC, Adan RA, Loos RJ, Onland-Moret NC, Grobbee DE, et al. Obesity genes identified in genome-wide association studies are associated with adiposity measures and potentially with nutrient-specific food preference. The American Journal of Clinical Nutrition. 2009;90(4):951–959. doi: 10.3945/ajcn.2009.27781 19692490

[10] Burt JV, Hertzler AA. Parental influence on the child’s food preference. Journal of Nutrition Education. 1978;10(3):127–128. doi: 10.1016/S0022-3182(78)80057-0

[11] Wisotsky Z, Medina A, Freeman E, Dahanukar A. Evolutionary differences in food preference rely on Gr64e, a receptor for glycerol. Nature Neuroscience. 2011;14(12):1534–1541. doi: 10.1038/nn.2944 22057190

[12] Provenza FD. Postingestive Feedback as an Elementary Determinant of Food Preference and Intake in Ruminants. Journal of Range Management. 1995;48(1):2. doi: 10.2307/4002498

[13] Adam CL, Williams PA, Dalby MJ, Garden K, Thomson LM, Richardson AJ, et al. Different types of soluble fermentable dietary fibre decrease food intake, body weight gain and adiposity in young adult male rats. Nutrition & Metabolism. 2014;11(1):36. doi: 10.1186/1743-7075-11-36 25152765 PMC4141268

[14] Roberts SB, Heyman MB. Dietary Composition and Obesity: Do We Need to Look beyond Dietary Fat? The Journal of Nutrition. 2000;130(2):267S–267S. doi: 10.1093/jn/130.2.267S 10721884

[15] Cani PD, Lecourt E, Dewulf EM, Sohet FM, Pachikian BD, Naslain D, et al. Gut microbiota fermentation of prebiotics increases satietogenic and incretin gut peptide production with consequences for appetite sensation and glucose response after a meal. The American Journal of Clinical Nutrition. 2009;90(5):1236–1243. doi: 10.3945/ajcn.2009.28095 19776140

[16] Yadav H, Lee JH, Lloyd J, Walter P, Rane SG. Beneficial Metabolic Effects of a Probiotic via Butyrate-induced GLP-1 Hormone Secretion. Journal of Biological Chemistry. 2013;288(35):25088–25097. doi: 10.1074/jbc.M113.452516 23836895 PMC3757173

[17] Medawar E, Haange SB, Rolle-Kampczyk U, Engelmann B, Dietrich A, Thieleking R, et al. Gut microbiota link dietary fiber intake and short-chain fatty acid metabolism with eating behavior. Translational Psychiatry. 2021;11(1):500. doi: 10.1038/s41398-021-01620-3 34599144 PMC8486801

[18] Gupta A, Osadchiy V, Mayer EA. Brain-gut-microbiome interactions in obesity and food addiction. Nature Reviews Gastroenterology & Hepatology. 2020;17(11):655–672. doi: 10.1038/s41575-020-0341-5 32855515 PMC7841622

[19] Finlayson G, King N, Blundell JE. Is it possible to dissociate ‘liking’ and ‘wanting’ for foods in humans? A novel experimental procedure. Physiology & Behavior. 2007;90(1):36–42. doi: 10.1016/j.physbeh.2006.08.020 17052736

[20] Steptoe A, Pollard TM, Wardle J. Development of a Measure of the Motives Underlying the Selection of Food: the Food Choice Questionnaire. Appetite. 1995;25(3):267–284. doi: 10.1006/appe.1995.0061 8746966

[21] Glenn Stanley B, Kyrkouli SE, Lampert S, Leibowitz SF. Neuropeptide Y chronically injected into the hypothalamus: A powerful neurochemical inducer of hyperphagia and obesity. Peptides. 1986;7(6):1189–1192. doi: 10.1016/0196-9781(86)90149-X 3470711

[22] Nagai K, Thibault L, Nishikawa K, Hashida A, Ootani K, Nakagawa H. Effect of glucagon in macronutrient self-selection: Glucagon-enhanced protein intake. Brain Research Bulletin. 1991;27(3-4):409–415. doi: 10.1016/0361-9230(91)90134-6 1959038

[23] Dickson PR, Vaccarino FJ. GRF-induced feeding: Evidence for protein selectivity and opiate involvement. Peptides. 1994;15(8):1343–1352. doi: 10.1016/0196-9781(94)90107-4 7700837

[24] Dickson PR, Feifel D, Vaccarino FJ. Blockade of endogenous GRF at dark onset selectively suppresses protein intake. Peptides. 1995;16(1):7–9. doi: 10.1016/0196-9781(94)00153-W 7716077

[25] Vaccarino FJ, Taube MR. Intra-Arcuate Opiate Actions Stimulate GRF-Dependent and Protein-Selective Feeding. Peptides. 1997;18(2):197–205. doi: 10.1016/S0196-9781(96)00283-5 9149291

[26] Tempel DL, Leibowitz KJ, Leibowitz SF. Effects of PVN galanin on macronutrient selection. Peptides. 1988;9(2):309–314. doi: 10.1016/0196-9781(88)90265-3 2453854

[27] Shor-Posner G, Grinker JA, Marinescu C, Brown O, Leibowitz SF. Hypothalamic serotonin in the control of meal patterns and macronutrient selection. Brain Research Bulletin. 1986;17(5):663–671. doi: 10.1016/0361-9230(86)90198-X 3801928

[28] Yano JM, Yu K, Donaldson GP, Shastri GG, Ann P, Ma L, et al. Indigenous Bacteria from the Gut Microbiota Regulate Host Serotonin Biosynthesis. Cell. 2015;161(2):264–276. doi: 10.1016/j.cell.2015.02.047 25860609 PMC4393509

[29] Matt SM, Allen JM, Lawson MA, Mailing LJ, Woods JA, Johnson RW. Butyrate and Dietary Soluble Fiber Improve Neuroinflammation Associated With Aging in Mice. Frontiers in Immunology. 2018;9:1832. doi: 10.3389/fimmu.2018.01832 30154787 PMC6102557

[30] Reed DR, Friedman MI, Tordoff MG. Experience with a macronutrient source influences subsequent macronutrient selection. Appetite. 1992;18(3):223–232. doi: 10.1016/0195-6663(92)90199-G 1510464

[31] Lozupone C, Lladser ME, Knights D, Stombaugh J, Knight R. UniFrac: an effective distance metric for microbial community comparison. The ISME Journal. 2011;5(2):169–172. doi: 10.1038/ismej.2010.133 20827291 PMC3105689

[32] Parnell JA, Reimer RA. Prebiotic fibres dose-dependently increase satiety hormones and alter Bacteroidetes and Firmicutes in lean and obese JCR:LA-cp rats. British Journal of Nutrition. 2012;107(4):601–613. doi: 10.1017/S0007114511003163 21767445 PMC3827017

[33] Li ETS, Anderson GH. Meal composition influences subsequent food selection in the young rat. Physiology & Behavior. 1982;29(5):779–783. doi: 10.1016/0031-9384(82)90324-9 7156215

[34] Singh A, Zapata RC, Pezeshki A, Reidelberger RD, Chelikani PK. Inulin fiber dose-dependently modulates energy balance, glucose tolerance, gut microbiota, hormones and diet preference in high-fat-fed male rats. The Journal of Nutritional Biochemistry. 2018;59:142–152. doi: 10.1016/j.jnutbio.2018.05.017 30005919

[35] Beisner J, Filipe Rosa L, Kaden-Volynets V, Stolzer I, Günther C, Bischoff SC. Prebiotic Inulin and Sodium Butyrate Attenuate Obesity-Induced Intestinal Barrier Dysfunction by Induction of Antimicrobial Peptides. Frontiers in Immunology. 2021;12:678360. doi: 10.3389/fimmu.2021.678360 34177920 PMC8226265

[36] Koch F, Derno M, Langhammer M, Tuchscherer A, Hammon HM, Mielenz M, et al. A high-protein diet containing inulin/oligofructose supports body weight gain associated with lower energy expenditure and carbohydrate oxidation, and alters faecal microbiota in C57BL/6 mice. Journal of Nutritional Science. 2021;10:e50. doi: 10.1017/jns.2021.42 34290864 PMC8278163

[37] Pauly MJ, Rohde JK, John C, Evangelakos I, Koop AC, Pertzborn P, et al. Inulin Supplementation Disturbs Hepatic Cholesterol and Bile Acid Metabolism Independent from Housing Temperature. Nutrients. 2020;12(10):3200. doi: 10.3390/nu12103200 33092056 PMC7589137

[38] Petersen A, Heegaard PM, Pedersen AL, Andersen JB, Sørensen RB, Frøkiær H, et al. Some putative prebiotics increase the severity of Salmonella entericaserovar Typhimurium infection in mice. BMC Microbiology. 2009;9(1):245. doi: 10.1186/1471-2180-9-245 19948011 PMC2789089

[39] Isken F, Klaus S, Osterhoff M, Pfeiffer AFH, Weickert MO. Effects of long-term soluble vs. insoluble dietary fiber intake on high-fat diet-induced obesity in C57BL/6J mice. The Journal of Nutritional Biochemistry. 2010;21(4):278–284. doi: 10.1016/j.jnutbio.2008.12.012 19369060

[40] Weitkunat K, Schumann S, Petzke KJ, Blaut M, Loh G, Klaus S. Effects of dietary inulin on bacterial growth, short-chain fatty acid production and hepatic lipid metabolism in gnotobiotic mice. The Journal of Nutritional Biochemistry. 2015;26(9):929–937. doi: 10.1016/j.jnutbio.2015.03.010 26033744

[41] Avena NM, Rada P, Hoebel BG. Sugar and Fat Bingeing Have Notable Differences in Addictive-like Behavior. The Journal of Nutrition. 2009;139(3):623–628. doi: 10.3945/jn.108.097584 19176748 PMC2714381

[42] Bello NT, Sweigart KL, Lakoski JM, Norgren R, Hajnal A. Restricted feeding with scheduled sucrose access results in an upregulation of the rat dopamine transporter. American Journal of Physiology-Regulatory, Integrative and Comparative Physiology. 2003;284(5):R1260–R1268. doi: 10.1152/ajpregu.00716.2002 12521926

[43] Colantuoni C, Schwenker J, McCarthy J, Rada P, Ladenheim B, Cadet JL, et al. Excessive sugar intake alters binding to dopamine and mu-opioid receptors in the brain:. Neuroreport. 2001;12(16):3549–3552. doi: 10.1097/00001756-200111160-00035 11733709

[44] Cottone P, Sabino V, Steardo L, Zorrilla EP. Opioid-Dependent Anticipatory Negative Contrast and Binge-Like Eating in Rats with Limited Access to Highly Preferred Food. Neuropsychopharmacology. 2008;33(3):524–535. doi: 10.1038/sj.npp.1301430 17443124

[45] Bocarsly ME, Berner LA, Hoebel BG, Avena NM. Rats that binge eat fat-rich food do not show somatic signs or anxiety associated with opiate-like withdrawal: Implications for nutrient-specific food addiction behaviors. Physiology & Behavior. 2011;104(5):865–872. doi: 10.1016/j.physbeh.2011.05.018 21635910 PMC3480195

[46] Wojnicki FHE, Stine JG, Corwin RLW. Liquid sucrose bingeing in rats depends on the access schedule, concentration and delivery system. Physiology & Behavior. 2007;92(4):566–574. doi: 10.1016/j.physbeh.2007.05.002 17612580

[47] Ifland JR, Preuss HG, Marcus MT, Rourke KM, Taylor WC, Burau K, et al. Refined food addiction: A classic substance use disorder. Medical Hypotheses. 2009;72(5):518–526. doi: 10.1016/j.mehy.2008.11.035 19223127

[48] Zilberter T. Food Addiction and Obesity: Do Macronutrients Matter? Frontiers in Neuroenergetics. 2012;4. doi: 10.3389/fnene.2012.00007 22661943 PMC3362736

[49] Pessoa J, Belew GD, Barroso C, Egas C, Jones JG. The Gut Microbiome Responds Progressively to Fat and/or Sugar-Rich Diets and Is Differentially Modified by Dietary Fat and Sugar. Nutrients. 2023;15(9):2097. doi: 10.3390/nu15092097 37432234 PMC10180990

[50] Then CK, Paillas S, Wang X, Hampson A, Kiltie AE. Association of Bacteroides acidifaciens relative abundance with high-fibre diet-associated radiosensitisation. BMC Biology. 2020;18(1):102. doi: 10.1186/s12915-020-00836-x 32811478 PMC7437060

[51] Kawasoe J, Uchida Y, Kawamoto H, Miyauchi T, Watanabe T, Saga K, et al. Propionic Acid, Induced in Gut by an Inulin Diet, Suppresses Inflammation and Ameliorates Liver Ischemia and Reperfusion Injury in Mice. Frontiers in Immunology. 2022;13:862503. doi: 10.3389/fimmu.2022.862503 35572528 PMC9097600

[52] Minokoshi Y, Nakajima K, Okamoto S. Homeostatic *versus* hedonic control of carbohydrate selection. The Journal of Physiology. 2020;598(18):3831–3844. doi: 10.1113/JP280066 32643799

[53] Agustí A, Campillo I, Balzano T, Benítez-Páez A, López-Almela I, Romaní-Pérez M, et al. Bacteroides uniformis CECT 7771 Modulates the Brain Reward Response to Reduce Binge Eating and Anxiety-Like Behavior in Rat. Molecular Neurobiology. 2021;58(10):4959–4979. doi: 10.1007/s12035-021-02462-2 34228269 PMC8497301

[54] Dong TS, Gupta A, Jacobs JP, Lagishetty V, Gallagher E, Bhatt RR, et al. Improvement in Uncontrolled Eating Behavior after Laparoscopic Sleeve Gastrectomy Is Associated with Alterations in the Brain-Gut-Microbiome Axis in Obese Women. Nutrients. 2020;12(10):2924. doi: 10.3390/nu12102924 32987837 PMC7599899

[55] Reigstad CS, Salmonson CE, Iii JFR, Szurszewski JH, Linden DR, Sonnenburg JL, et al. Gut microbes promote colonic serotonin production through an effect of short‐chain fatty acids on enterochromaffin cells. The FASEB Journal. 2015;29(4):1395–1403. doi: 10.1096/fj.14-259598 25550456 PMC4396604

[56] Magnusson KR, Hauck L, Jeffrey BM, Elias V, Humphrey A, Nath R, et al. Relationships between diet-related changes in the gut microbiome and cognitive flexibility. Neuroscience. 2015;300:128–140. doi: 10.1016/j.neuroscience.2015.05.016 25982560

[57] Sen T, Cawthon CR, Ihde BT, Hajnal A, DiLorenzo PM, De La Serre CB, et al. Diet-driven microbiota dysbiosis is associated with vagal remodeling and obesity. Physiology & Behavior. 2017;173:305–317. doi: 10.1016/j.physbeh.2017.02.027 28249783 PMC5428886

[58] Suriano F, Vieira-Silva S, Falony G, De Wouters d’Oplinter A, Paone P, Delzenne NM, et al. Fat and not sugar as the determining factor for gut microbiota changes, obesity, and related metabolic disorders in mice. American Journal of Physiology-Endocrinology and Metabolism. 2023;324(1):E85–E96. doi: 10.1152/ajpendo.00141.2022 36516223

[59] Fan Y, Støving RK, Berreira Ibraim S, Hyötyläinen T, Thirion F, Arora T, et al. The gut microbiota contributes to the pathogenesis of anorexia nervosa in humans and mice. Nature Microbiology. 2023;8(5):787–802. doi: 10.1038/s41564-023-01355-5 37069399 PMC10159860

