# Supplementary figures and images for: Dietary fiber induces a fat preference associated with the gut microbiota
Source: PLoS One. 2024 Jul 10;19(7):e0305849. doi: 10.1371/journal.pone.0305849 (PMC11236109; doi:10.1371/journal.pone.0305849)

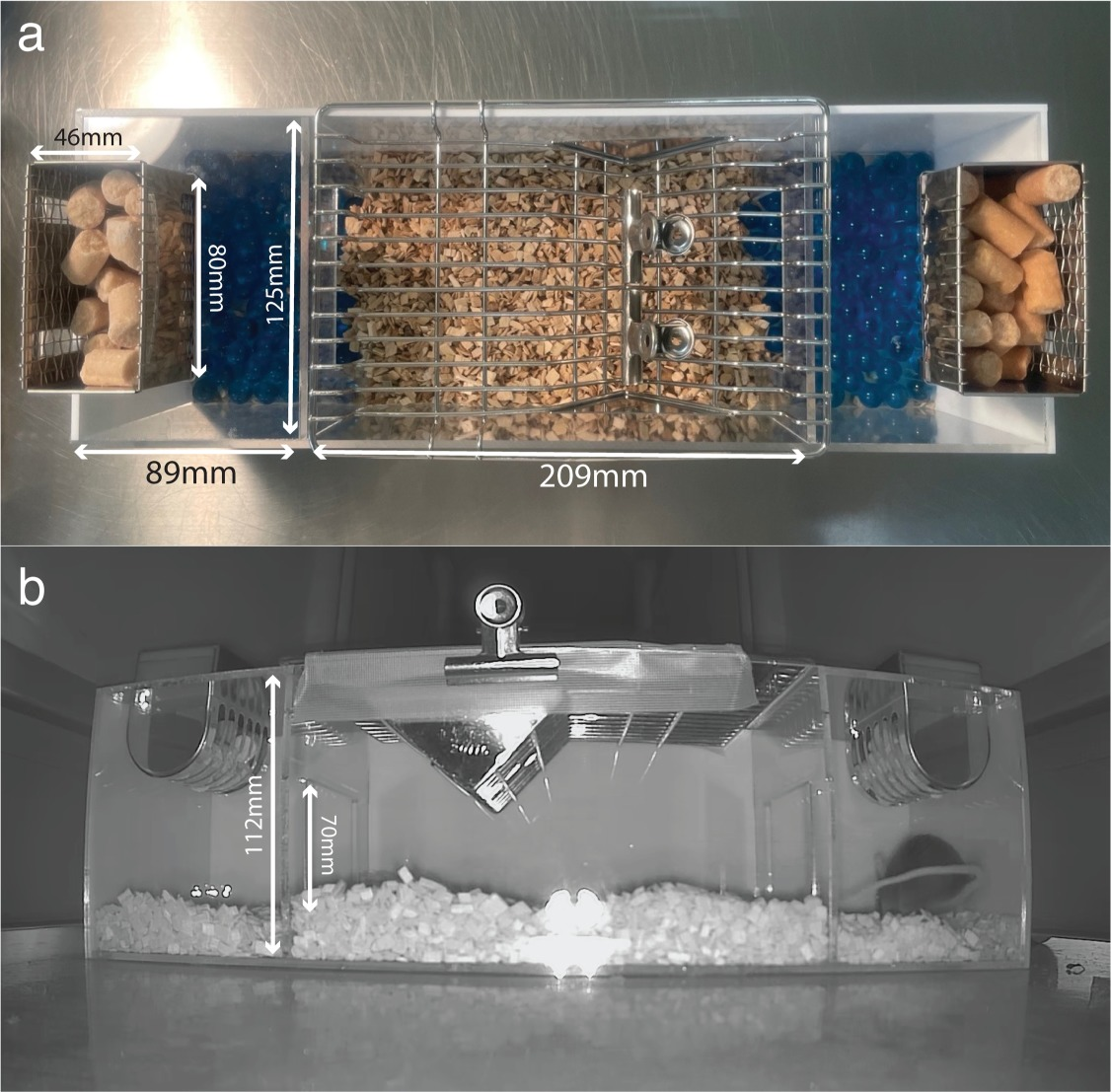

Supplement: S1 Fig — The left- and right-chambers are the feeding chambers for mice to self-select between the test diets; the central area is the resting area for when the mice are not interacting with food. Marble balls are modifications made to the cage to prevent the mice from resting in the feeding chambers; stainless steel clips are used to secure the lid to prevent the mice from escaping. (TIF) [file pone.0305849.s001.tif]

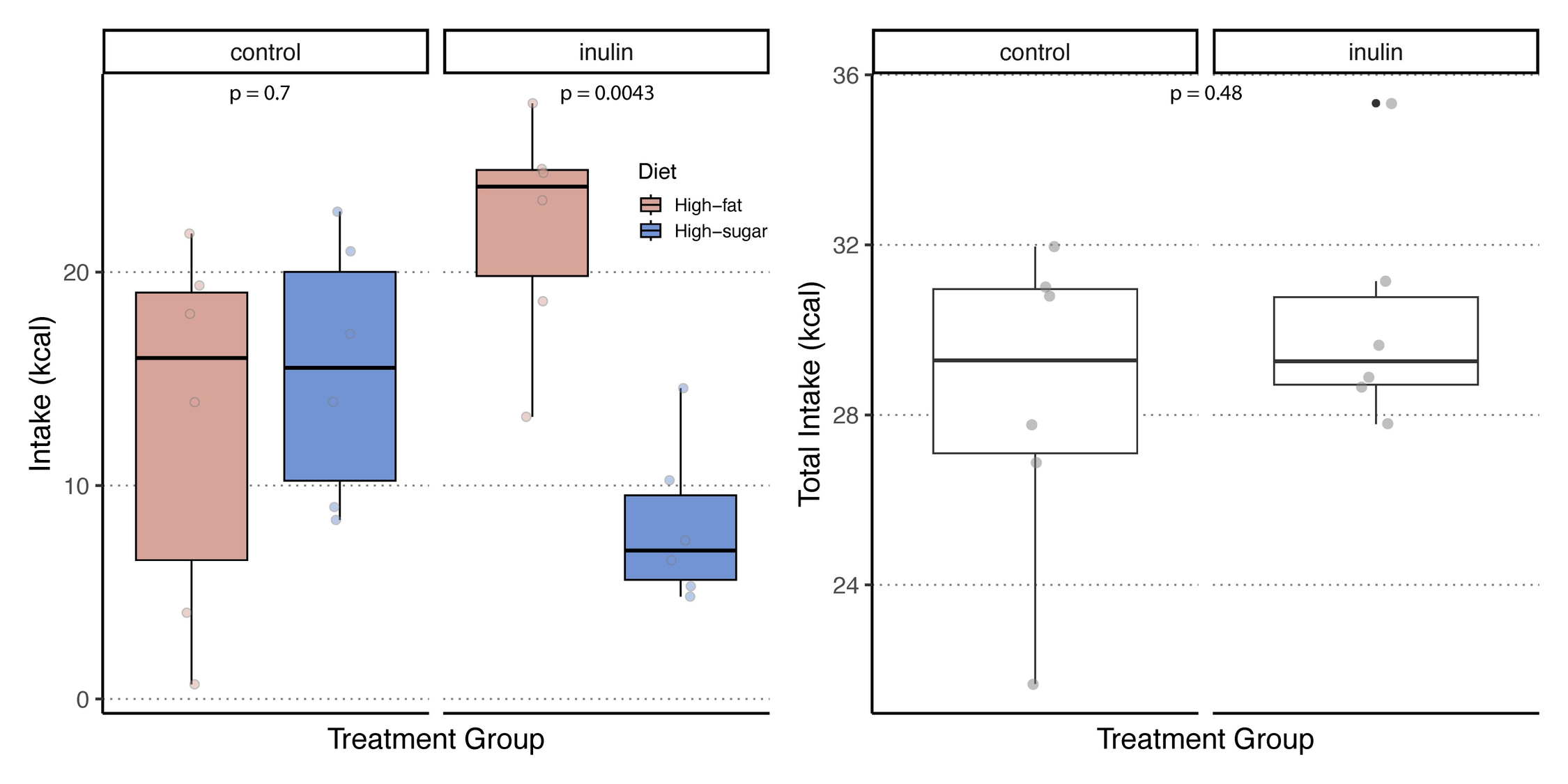

Supplement: S2 Fig — Fig 3A: No significant differences were detected in the energy intake of high-fat and high-sugar test diet energy intakes among the inulin-conditioned and control mice (P = 0.7 and 0.13, respectively). Fig 3B: Total energy intake combining high-fat and high-sugar test diets of the inulin-conditioned and control mice; no significant differences were detected in the total energy intake between the two groups (P = 0.66). (TIF) [file pone.0305849.s002.tif]

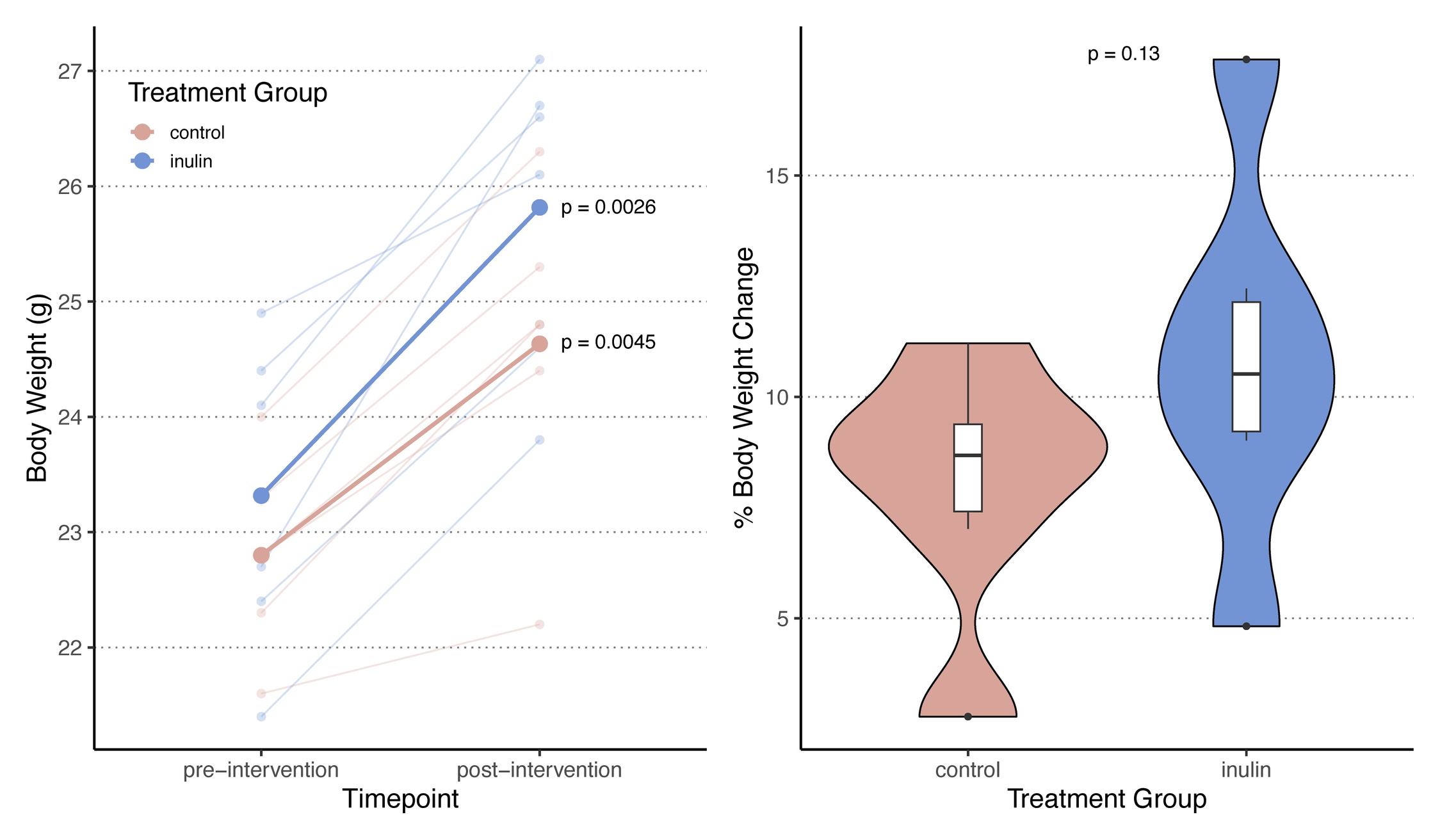

Supplement: S3 Fig — The left panel illustrates the body weight of mice from control and inulin-conditioned groups at two distinct time points: pre-intervention (baseline) and post-intervention. Each line represents the weight trajectory of an individual mouse. The right panel visualizes the percentage change in body weight for each group. The difference in weight gain between the two groups was not statistically significant (P = 0.229). (TIF) [file pone.0305849.s003.tif]

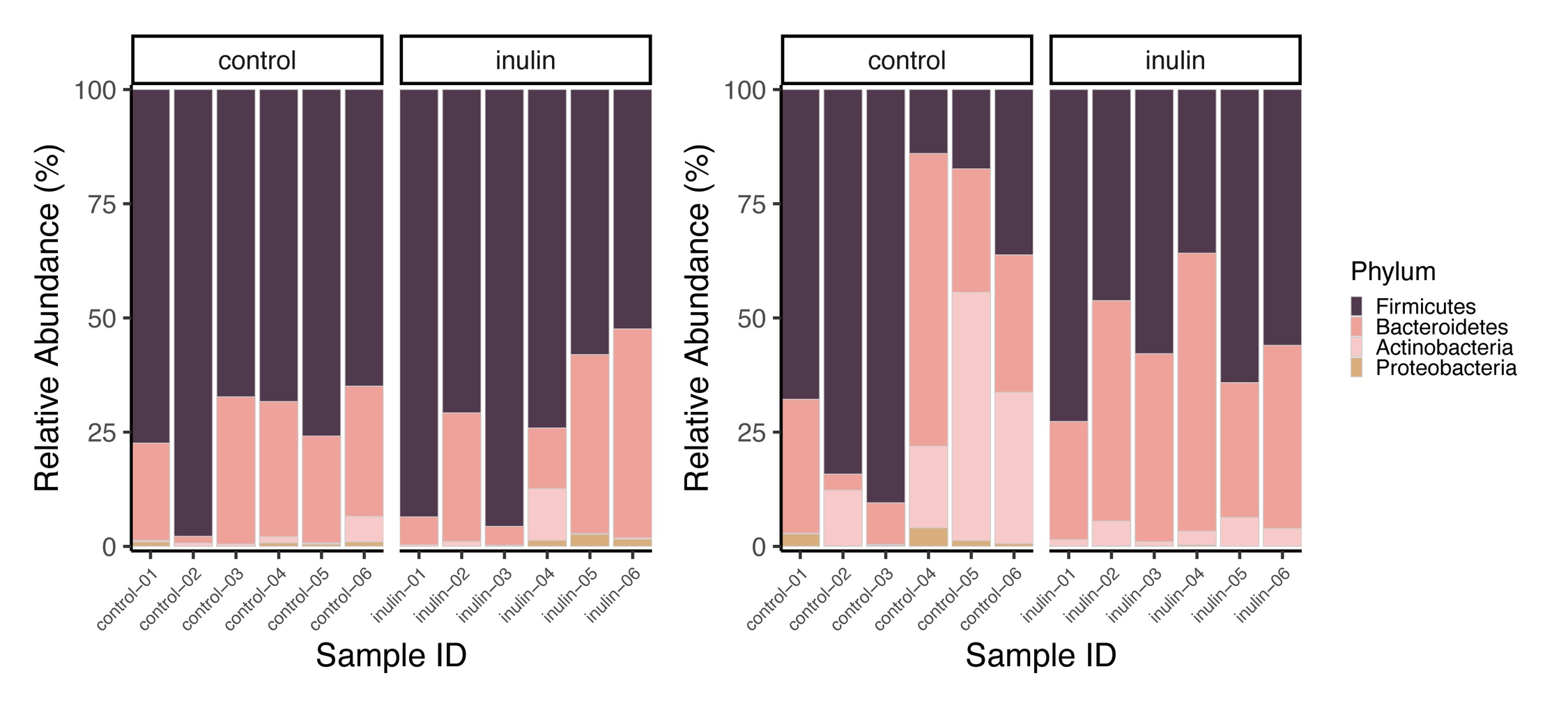

Supplement: S4 Fig — The figure illustrates the relative abundance of different bacterial phyla within each sample group (control and inulin-conditioned) at both the pre- and post-intervention stages, highlighting shifts in microbial diversity in response to the intervention. (TIF) [file pone.0305849.s004.tif]

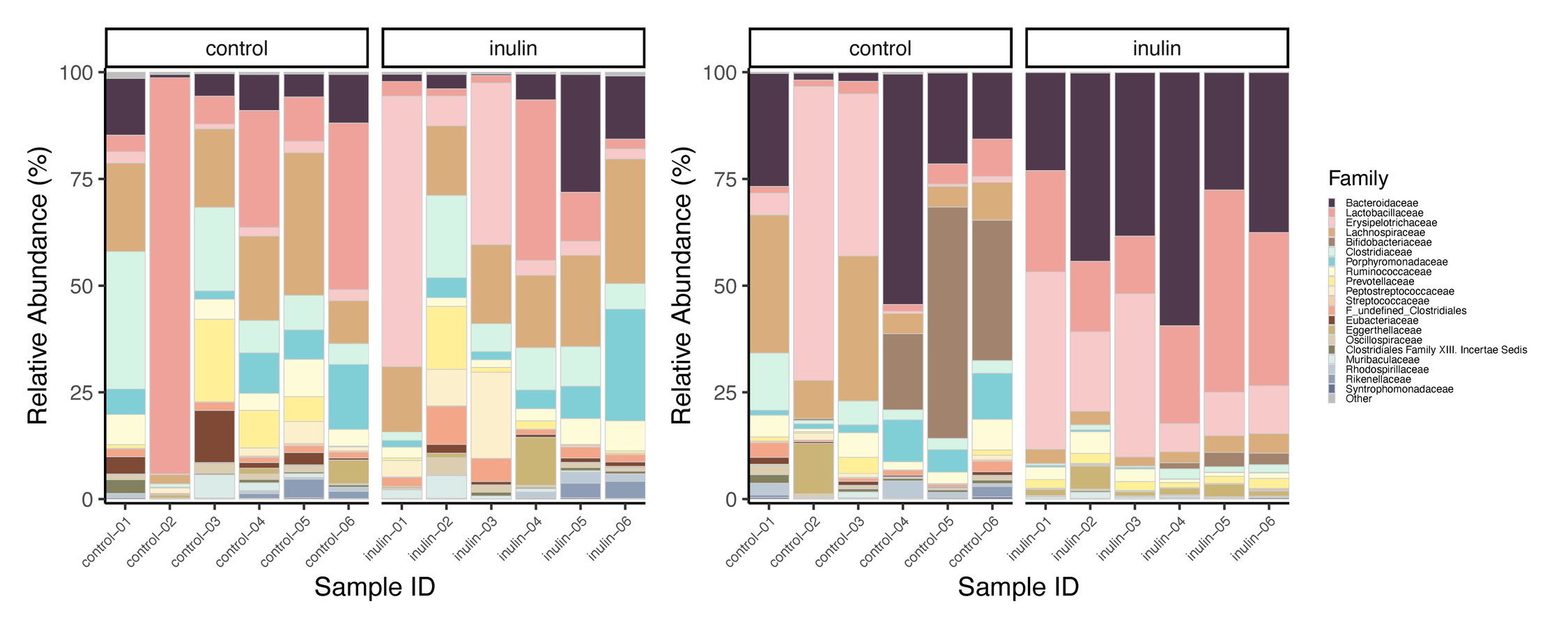

Supplement: S5 Fig — The figure illustrates the relative abundance of different bacterial phyla within each sample group (control and inulin-conditioned) at both the pre- and post-intervention stages, highlighting shifts in microbial diversity in response to the intervention. (TIF) [file pone.0305849.s005.tif]

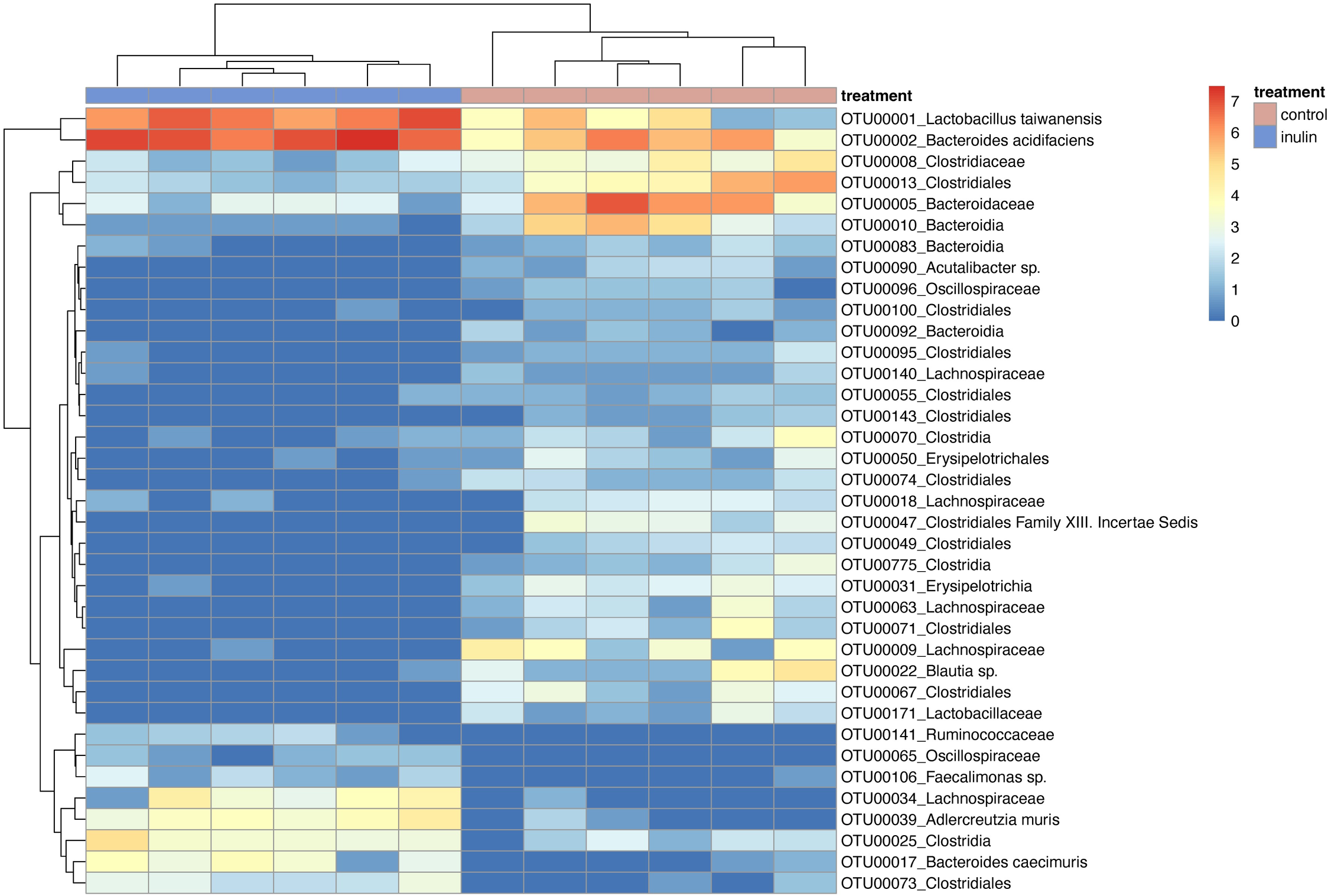

Supplement: S6 Fig — The colors on the heatmap reflect the log-transformed OTU relative abundance; red indicates OTUs high in relative abundance and blue indicates OTUs low in relative abundance. (TIF) [file pone.0305849.s006.tif]

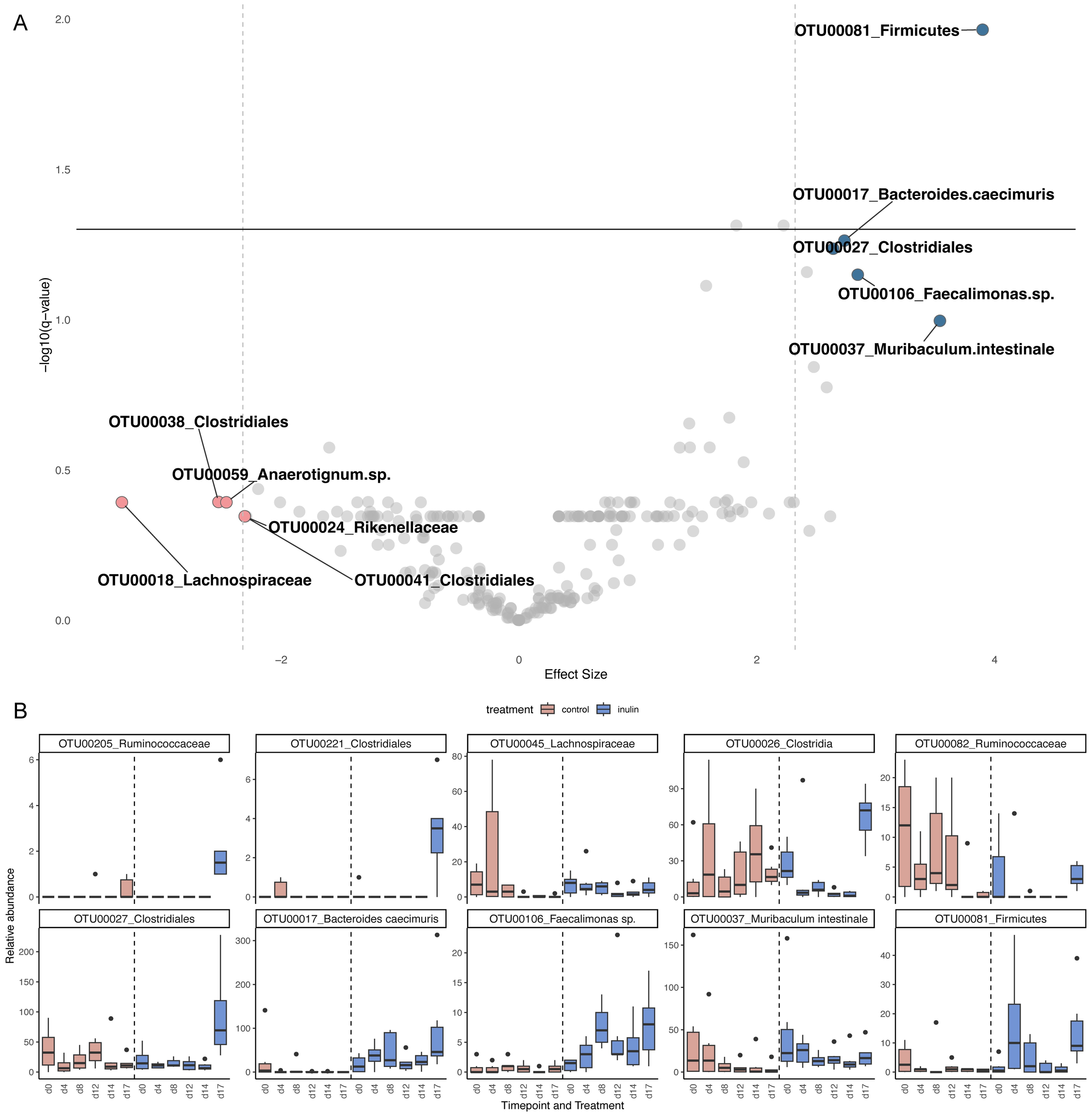

Supplement: S7 Fig — S5A Fig shows a volcano plot showcasing bacterial taxa that exhibited significant differences in relative abundance following a preference test, as analyzed by MaAsLin2. The top 10 taxa with the most pronounced differences are highlighted with labels. Supplementary S5B Fig provides a longitudinal assessment of the relative abundance of these top taxa at distinct time points (Days 0, 4, 8, 12, and 14). (TIF) [file pone.0305849.s007.tif]

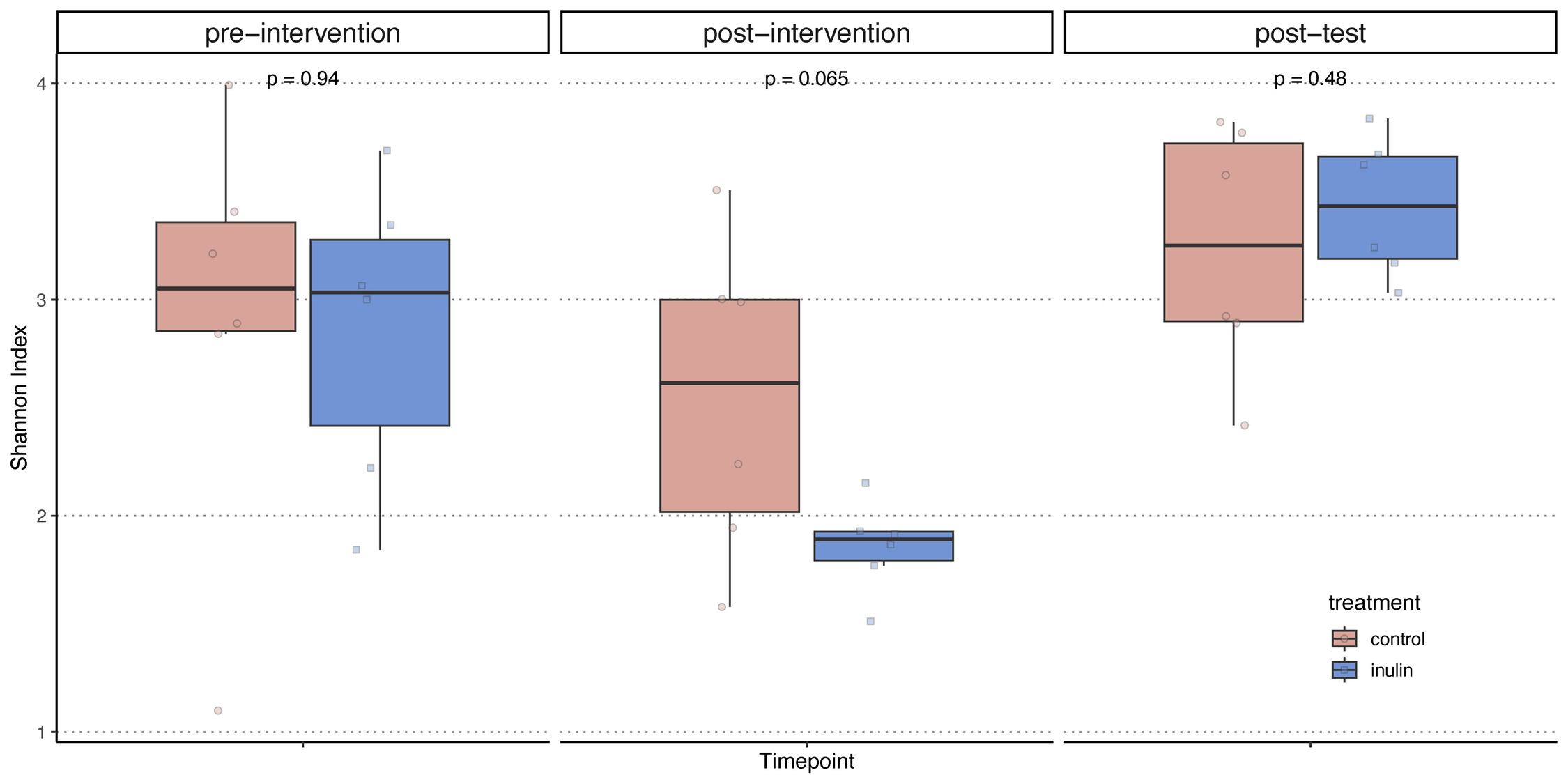

Supplement: S8 Fig — S6 Fig illustrates the α-diversity (Shannon diversity index) of microbial communities for the control and inulin-conditioned groups at three distinct time points: pre-intervention, post-intervention, and post-preference tests were not significantly different between the control and inulin-conditioned mice (P = 0.94, 0.065, 0.48, respectively). (TIF) [file pone.0305849.s008.tif]

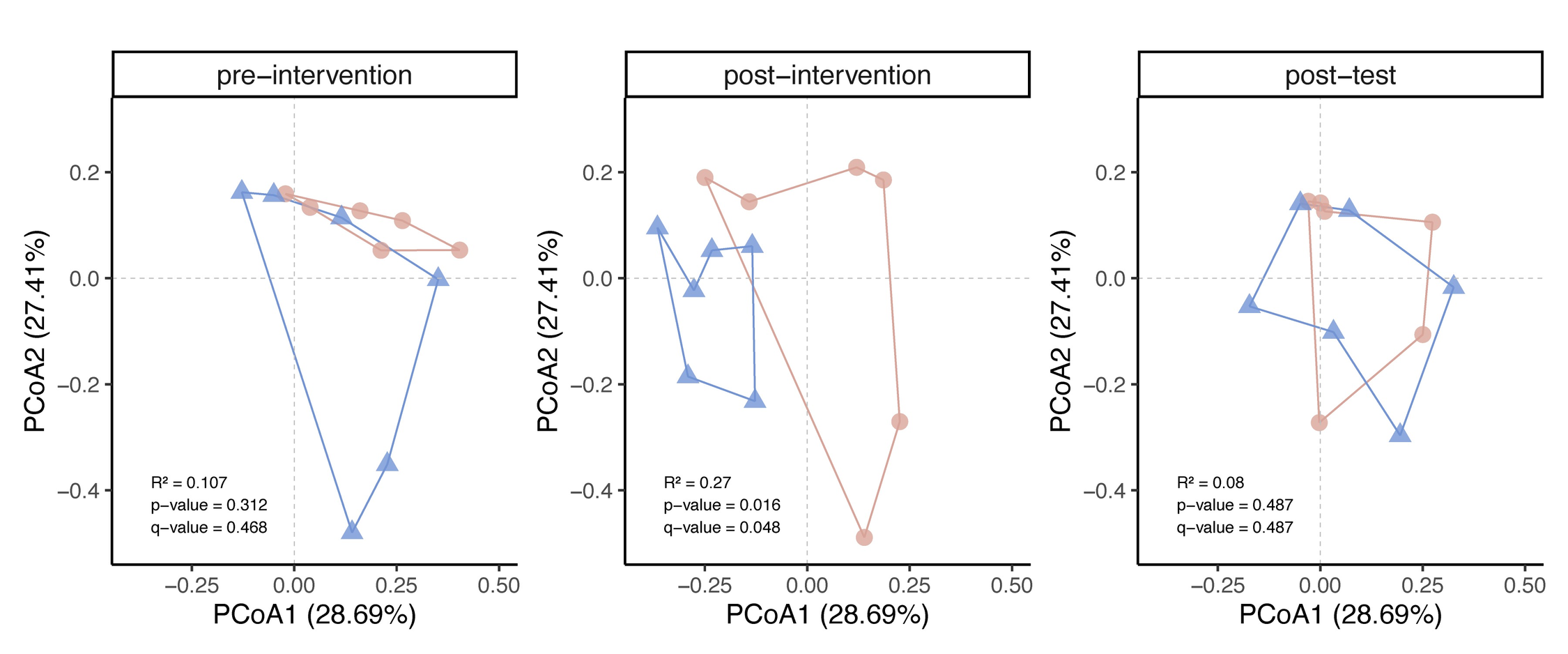

Supplement: S9 Fig — S7 Fig showcases microbial community dissimilarity during three stages: pre-intervention, post-intervention, and post-test. Gut microbiota profiles were significantly different between the control and inulin-conditioned mice at the post-intervention time point (R2 = 0.27, q-value = 0.048), but not at the pre-intervention (R2 = 0.107, q-value = 0.468) or post-preference test (R2 = 0.08, q-value = 0.487) time points. (TIF) [file pone.0305849.s009.tif]
